# Casein Kinase 1 Proteomics Reveal Prohibitin 2 Function in Molecular Clock

**DOI:** 10.1371/journal.pone.0031987

**Published:** 2012-02-27

**Authors:** Lorna S. Kategaya, Aisha Hilliard, Louying Zhang, John M. Asara, Louis J. Ptáček, Ying-Hui Fu

**Affiliations:** 1 Department of Neurology, University of California San Francisco, San Francisco, California, United States of America; 2 Howard Hughes Medical Institute, University of California San Francisco, San Francisco, California, United States of Ameica; 3 Division of Signal Transduction, Beth Israel Deaconess Medical Center and Department of Medicine, Harvard Medical School, Boston, Massachusetts, United States of America; Yale School of Medicine, United States of America

## Abstract

Throughout the day, clock proteins synchronize changes in animal physiology (e.g., wakefulness and appetite) with external cues (e.g., daylight and food). In vertebrates, both casein kinase 1 delta and epsilon (CK1δ and CK1ε) regulate these circadian changes by phosphorylating other core clock proteins. In addition, CK1 can regulate circadian-dependent transcription in a non-catalytic manner, however, the mechanism is unknown. Furthermore, the extent of functional redundancy between these closely related kinases is debated. To further advance knowledge about CK1δ and CK1ε mechanisms of action in the biological clock, we first carried out proteomic analysis of both kinases in human cells. Next, we tested interesting candidates in a cell-based circadian readout which resulted in the discovery of PROHIBITIN 2 (PHB2) as a modulator of period length. Decreasing the expression of PHB2 increases circadian-driven transcription, thus revealing PHB2 acts as an inhibitor in the molecular clock. While stable binding of PHB2 to either kinase was not detected, knocking down CK1ε expression increases PHB2 protein levels and, unexpectedly, knocking down CK1δ decreases PHB2 transcript levels. Thus, isolating CK1 protein complexes led to the identification of PHB2 as an inhibitor of circadian transcription. Furthermore, we show that CK1δ and CK1ε differentially regulate the expression of PHB2.

## Introduction

All vertebrates have orthologues for casein kinase 1 isoforms, delta and epsilon (CK1δ and CK1ε), which play prominent roles in diverse cellular processes such as proliferation, growth, and survival [1]. CK1δ/ε regulate daily biological rhythms as part of the core molecular clock [2]. In humans, point mutations in both CK1δ (NP_001884) and its substrate, PERIOD 2 (PER2 (NP_073728)), have been shown to cause familial advanced sleep phase syndrome [3], [4]. PER2 is a prominent protein component of the negative feedback loop necessary for the daily cycling of gene transcripts [2]. PERs and CRYPTOCHROMES (CRYs) inhibit BMAL (NP_001001463) and CLOCK (NP_004889) driven transcription of genes that contain E-box response elements, including *PER* and *CRY*. Systematic control of transcription, translation and post-translation modifications is essential to efficiently regulate physiological changes (e.g. body temperature and metabolism) that occur throughout the day.

In addition to enzymatic regulation of core clock proteins, recent work suggests that CK1 acts in a non-catalytic manner to regulate circadian rhythms [5]. We carried out proteomic analysis of CK1δ and ε to gain a better understanding of how they function in the molecular clock. Considering the inherent temporal nature of circadian biology, we isolated CK1 protein complexes in synchronized cells and tested interesting candidates in a circadian cell-based assay using dexamethasone, a synthetic glucocorticoid, to reset the molecular clock in peripheral cells [6].

## Results and Discussion

**Figure 1:**
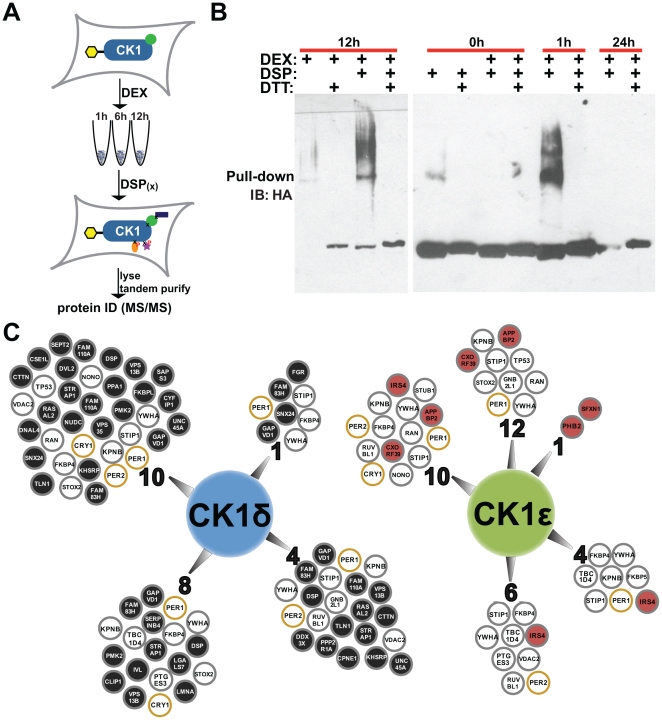
Time-dependent CK1δ and CK1ε proteomics. (**A**) Illustration of screen. Cells stably expressing dually-tagged (yellow hexagon) CK1 were treated with dexamethasone (DEX) and harvested at different time points. Protein complexes were stabilized with DSP prior to lysis and purification for peptide identification by LC-MS/MS. (**B**) HA immunoblot showing DSP efficacy and the effect of dexamethasone on CK1ε complexes. (**C**) Diagram showing proteins pulled out at the different time points by CK1δ and CK1ε. Numbers represent hours after DEX that cells were harvested. Open circles were pulled out with both CK1δ and CK1ε (orange circles are known circadian proteins). Closed circles were pulled out with CK1δ alone (black-fill) or CK1ε alone (red-fill).

The purpose of this study was to use CK1δ/ε proteomics to gain mechanistic insights into the molecular clock. TAPTag has been successfully used to study basal protein-protein interactions in various cell types by stably over-expressing low amounts of dually-tagged bait proteins [7]. We chose to isolate dexamethasone-dependent CK1 protein complexes over time due to the inherent temporal nature of circadian rhythms (Fig. 1A). Upon establishing that circadian gene oscillation occurs in HEK293 (HEK) cells synchronized with dexamethasone (Fig. S1A), we made HEK CK1δ- and ε-stable cell lines. To capture whole protein complexes including proteins bound to the kinases (directly (orthosterically and allosterically), indirectly, stably, and transiently), intact cells were treated with a cell-permeable thio-cleavable crosslinker, dithiobis[succinimidyl propionate] (DSP). DSP, a homobifunctional crosslinker, reacts with primary amines that are at most 12.0 Å apart [8]. Intracellular crosslinking maintains protein subcellular localization and increases retention of proteins that would otherwise be lost in the process of purification. To determine crosslinking efficacy, CK1ε (NP_001885) stable cells were treated with dexamethasone, harvested 12 h (hour) later, divided in half and treated with vehicle (DMSO) or DSP. Following tandem purification, 10% of the final elution was analyzed by Western blot (Fig. 1B). In non-reducing conditions, DSP-treated cells produced higher molecular weight bands containing CK1ε that were not detected in eluate from cells treated with vehicle (Fig. 1B, left). This result suggested that the crosslinker was working as expected to stabilize larger CK1ε protein complexes. Next, we investigated the effect of dexamethasone on these DSP-stabilized protein complexes immediately, 1 h and 24 h after dexamethasone exposure. As shown in Fig. 1B (right) under non-reducing conditions, there was no observable difference in the intensity of higher molecular weight bands from eluates of cells treated with vehicle or dexamethasone immediately following treatment. However, in cells harvested 1 h later, we observed an increase in CK1ε protein complexes. 24 h later, CK1ε expression is reduced, suggesting a decrease in the cell viability under these conditions. When we treated final eluates with the reducing agent DTT, we observed a single band of the expected size of tagged CK1ε in all conditions. Together, these results suggested that we could induce, stabilize and isolate dexamethasone-induced CK1 protein complexes. Thus, DSP-stabilized protein complexes were isolated using tandem affinity tagging (TAPTag) purification at time points between 1 and 12 hours followed by protein identification using tandem mass spectrometry (LC-MS/MS).

Following the protocol outlined above, peptides identified by LC-MS/MS (Fig. 1C (see Table S1 for complete list)) suggested that while there was considerable overlap in the proteins bound by CK1δ and CK1ε, there were also some differences. First, the similarities: we identified known core clock proteins, PER1 (NP_002607), PER2 and CRYPTOCHROME 1 (CRY1 (NP_004066)) and a PER1-associated protein, NON POU DOMAIN CONTAINING OCTAMER BINDING PROTEIN (NONO (NP_001138880)) [9]. Additionally, proto-oncogene P53 (TP53 (NP_000537)), a known substrate, and phospho-serine/threonine signaling molecules 14-3-3 proteins (YWHA (NP_003395)), were pulled out by both kinases. These results substantiated our method. With regard to differences in binding, we consistently pulled out two poorly studied proteins with CK1δ but not CK1ε, GAPVD1 (GTPASE-ACTIVATING PROTEIN AND VP59 CONTAINING PROTEIN 1 (NP_079985)) and FAM83H (NP_940890). While further analysis is needed to confirm the binding specificity of these proteins, our results further support the hypothesis that CK1δ and CK1ε do not act completely redundantly.

**Figure 2:**
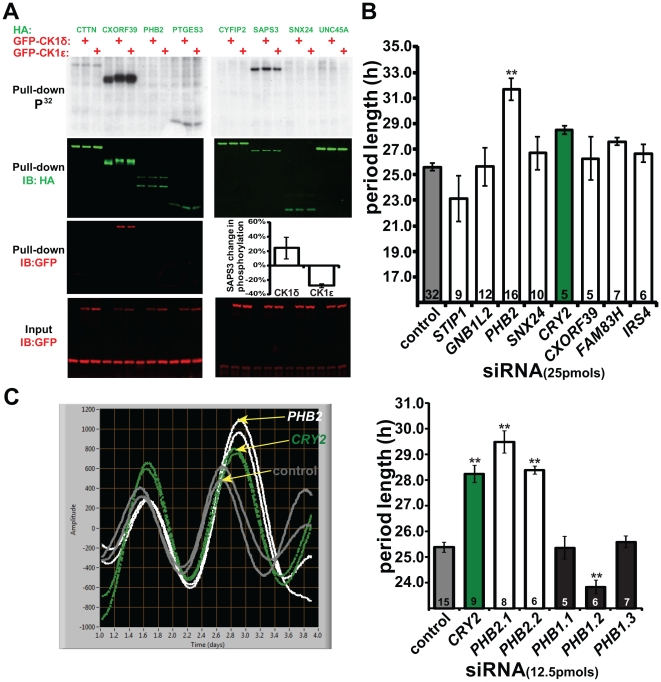
Cell-based kinase and Lumicycle assays. (**A**) Autoradiography (P^32^) and immunoblots of HA-tagged proteins (green) and GFP-tagged CK1δ or CK1ε (red). Graph *inset* showing percent change of SAPS3 phosphorylation by CK1δ and CK1ε (n = 2). (**B**) Results from LumiCycle assays in cells transfected with indicated siRNAs. (**C**) Representative traces after cell synchronization of control (gray), *CRY2* (green) and *PHB2* (white) siRNAs (***left***). Graph showing effects of control siRNA, *CRY2* siRNA, two *PHB2* siRNAs (*PHB*2.1 & *PHB*2.2) and three *PHB1* siRNAs (*PHB*1.1, *PHB*1.2, *PHB*1.3) (*right*). (Sample size indicated by number inside each bar, ** indicates p<0.001 following regression analysis. Error bars indicate SEM).

Upon pulling out known substrates, we next tested for novel CK1δ/ε binding partners and/or substrates using cell-based co-purification and kinase assays. Tagged-hits were transiently transfected either alone or with CK1δ or ε, after which the cells were incubated with radio-labeled sodium orthophosphate (P^32^) to detect phosphorylation. The overexpressed protein hits were purified and analyzed by Western Blot analysis. One of the eight proteins tested, CXORF39 (NP_997201), co-purified with both kinases (Fig. 2A). Furthermore, cotransfection of CXORF39 with GFP-CK1δ or GFP-CK1ε slowed its mobility (HA) and increased its P^32^ incorporation. This result suggests that CXORF39, a protein of unknown function, may be a novel CK1 substrate and binding partner. In contrast to the change in phosphorylation of CXORF39, we detected weaker changes in the phospho-state of SERINE/THREONINE-PROTEIN PHOSPHATASE 6 REGULATORY SUBUNIT 3 (SAPS3 (NP_997201)). Using densitometry to quantify SAPS3 P^32^ and HA bands, we determined the ratio of P^32^ to HA (Fig. 2A, *graph inset*) and detected a small increase with CK1δ, and interestingly, a decrease with CK1ε, in P^32^ incorporation. These results provide interesting leads for two proteins whose phospho-state was altered by increasing the expression levels of CK1δ and CK1ε. Further experiments including *in vitro* kinase assays with purified proteins and identification of the relevant sites of phosphorylation will be necessary to determine whether CXORF39 and SAPS3 are direct CK1 substrates.

Since the purpose of this study was to explore the molecular mechanisms underlying CK1δ and CK1ε clock function, we performed Lumicycle assays to test candidate proteins from proteomic results. In this circadian assay, U2OS cells that are stably expressing luciferase driven by the *BMAL1* promoter can be monitored over several days [10]. BMAL1 is a transcription factor that drives, and is regulated, in a circadian manner. Cells were transfected with siRNA of candidate genes, synchronized with dexamethasone, and cyclical bioluminescence was measured. We selected candidates to target with siRNA based on their potential roles in cell signaling. As shown in Figure 2B, knock-down of *PHB2* led to the most robust change (lengthening) of the period length. A second siRNA targeting *PHB2* also lengthened the period of reporter oscillation. Of the 3 siRNAs targeting PROHIBITIN (PHB1 (NP_002625)), a closely related protein that has been shown to heterodimerize with PHB2 (Fig. S1B and [11]), only one (*PHB*1.2) had an effect (Fig. 2C, right). Knock-down efficacy of the prohibitin siRNAs was confirmed using over-expressed proteins (Fig. S1C). While the other 2 *PHB1* siRNAs (*PHB*1.1 and *PHB*1.3) both effectively knocked down PHB1 protein levels, they did not affect period length. We thus interpret the *PHB1.2* siRNA result as a non-specific effect. Both PHB1 and PHB2 have previously been shown to regulate mitochondrial function and estrogen signaling [11]. Our findings suggest that specifically targeting PHB2 and not a closely related protein, PHB1, perturbs the normal regulation of circadian rhythmicity. Similar results were obtained when *Drosophila* adult locomotor activity was monitored following RNAi-facilitated knock-down of PHB1 and 2 (Table S2, Fig. S2 and S3). Additionally, we measured transcript levels of both *dprohibitins* in wild-type fly heads under constant darkness every 2 h for 48 h and found that they were not regulated in a circadian manner (Fig. S2A). This is consistent with previous results from mouse studies that are cataloged in the BIOGPS circadian database (http://biogps.org/circadian/#goto=genereport&id=11331).

**Figure 3:**
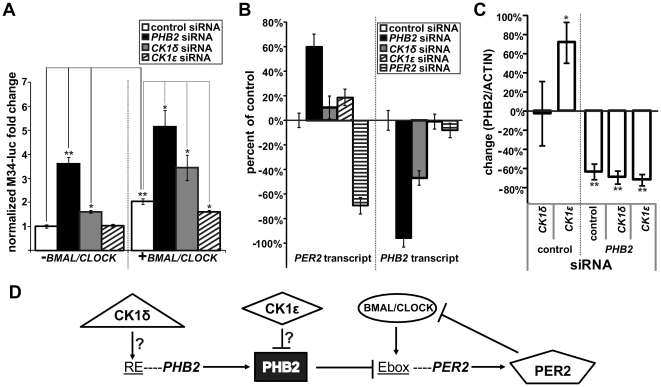
PHB2 is molecular clock component. (**A**) Representative graph (n = 3) of M34-luciferase reporter assays following transient cotransfections of M34-luc, renilla, GFP and indicated siRNAs without (left) or with (right) *BMAL1* and *CLOCK*. (**B**) Representative graph (n = 3) showing transcript levels of *PER2* and *PHB2* 24 h after transfecting cells with control, *PHB2*, *CK1δ*, *CK1ε* and *PER2* siRNA. (**C**) Graph (n = 3) showing the effect of indicated siRNAs without (left, marked as control) or with (right, marked as *PHB2*) *PHB2* siRNA on PHB2 protein levels relative to ACTIN. (**D**) Illustration summarizing molecular mechanism based on our results where shapes indicate proteins. (* indicates p<0.05; ** indicates p<0.01; determined by student t-test analysis. Error bars indicate STDEV).

To gain mechanistic insights into whether PHB2 regulates circadian gene expression, we carried out luciferase assays using the M34-luc reporter that contains multiple E-boxes upstream of luciferase [12]. BMAL1 and CLOCK bind to E-boxes in the promoters of clock controlled genes thus activating their transcription [2]. PHB2 loss-of-function (LOF) via siRNA, in the absence or presence of BMAL1/CLOCK, resulted in a significant increase in reporter activity compared to control (Fig. 3A). CK1δ LOF also potentiated reporter activity both in the absence and presence of over-expressed BMAL1/CLOCK. Conversely, CK1ε LOF significantly reduced BMAL1/CLOCK-activation of the reporter (p<0.05). Considering this result, we measured endogenous *PER2* mRNA by RT-qPCR following the knockdown of *PHB2*, *CK1δ* and *CK1ε*. PER2 is a prominent protein component of the negative feedback loop in the clock that oscillates in a circadian manner [2]. As predicted, decreasing *PHB2* expression resulted in an increase in *PER2* mRNA (Fig. 3B). We also observed the expected *PHB2* and *PER2* siRNA-facilitated decrease in their respective transcripts compared to control. Surprisingly, knocking down *CK1δ* led to a decrease in *PHB2*, suggesting that CK1δ plays a role in regulating *PHB2* transcription. Taken together, these results make a strong case for PHB2 acting as a repressor of E-box dependent transcription.

Lastly, we investigated whether CK1δ and CK1ε regulate the expression of endogenous PHB2 protein. Monitoring PHB2 relative to ACTIN (Fig. 3C) showed that CK1δ LOF had a variable effect while reducing CK1ε expression consistently resulted in an increase on PHB2 protein expression without altering *PHB2* mRNA levels (Fig. 3B). Notably, we initially pulled out PHB2 with CK1ε 1 h after dexamethasone treatment (Fig. 1C). Considering we cannot detect basal binding or a change in PHB2 phosphorylation state following the overexpression of CK1ε (Fig. 2A), it is likely that the effect of CK1ε on PHB2 protein may be indirect, requiring other protein(s). Thus, while *PHB2* transcription is controlled by CK1δ, its protein levels are regulated by CK1ε (Fig. 3C). Previous studies have shown that knocking down the expression of CK1δ and CK1ε (http://biogps.org/circadian/#goto=genereport&id=1454) led to lengthening of the circadian period in U2OS cells. Interestingly, we found that knocking down the expression of PHB2 also led to lengthening of the circadian period. Given our results showing PHB2 is regulated by CKIδ and CKIε at both mRNA and protein levels, it is possible that PHB2 acts, at least in part, as a downstream executor for the effects of CK1δ and/or CK1ε on circadian period.

In this study we have shown that PHB2 is new regulatory protein in the molecular clock. We discovered PHB2 using proteomics in search of potential substrates and binding partners of CK1δ and CK1ε (Fig. 1). Due to our specific interest in the circadian role of both kinases, we then narrowed our search using a secondary screen in human cells to identify which of these proteins were involved in circadian biology. As a result, we identified PHB2 as a regulator of circadian rhythms (Fig. 2C and S3). We show that the mechanism by which PHB2 carries out its function is by inhibiting circadian-driven transcription. Finally we found that CK1δ promotes *PHB2* transcription, whereas CK1ε reduces its protein expression (Fig. 3). However, we were unable to detect PHB2 bound to (or phosphorylated by) either kinase under basal conditions (Fig. 2A), indicating that CK1 regulates PHB2 indirectly.

Our study extends previous studies regarding PHB2 functioning independently from PHB1, an interaction that is crucial for the maintenance of mitochondrial morphology [11]. We show that reducing the expression of PHB2, but not PHB1, affects circadian rhythmicity in cells (Fig. 2C). PHB2 (but not PHB1) has been shown to translocate from the mitochondria to the nucleus in cells following treatment with estrogen to regulate gene transcription [13]. Additionally, another protein (BIG3/WRD5) has been shown to bind to and prevent translocation of PHB2 to the nucleus when overexpressed [14]. Interestingly, WRD5 is a PER1-associated protein that indirectly regulates circadian gene transcription [9] providing another clue on how PHB2 may function. Future molecular studies and functional assays examining whether CK1δ, CK1ε, PHB2 and WRD5 act together to regulate circadian biology are necessary.

In summary, we have shown that PHB2 is novel regulatory protein in the molecular clock by isolating CK1δ and CK1ε protein complexes. Studies involving kinases often focus on identifying protein substrates [15]. While kinase-substrate interactions mechanistically explain several kinase-dependent cellular outcomes, kinase function relies on constantly changing auxiliary protein-protein interactions. The major strength of this study is that we examined whole protein complexes in a context-dependent manner. Dynamic proteomics coupled with relevant molecular readouts will advance knowledge not only about normal protein function but can also provide drug targets for relevant human disease states. This feasible approach is especially relevant when elucidating how single proteins such as CK1δ and CK1ε carry out their pleiotropic roles.

## Materials and Methods

### Plasmids and antibodies

PGLUE vector was used to clone TAPTAG constructs that were used for proteomics and kinase assays. GFP constructs were made in a pCDNA3 vector background. A rat HA antibody (3F10) was used to blot for HA in the pGLUE constructs. Rabbit anti-human PHB2 antibody was obtained from Santa Cruz (sc-67045). Mouse anti-ACTIN antibody was obtained from Sigma (A5316). Secondary antibodies were obtained from LICOR Biosciences.

### TAP-Tag with DSP and dexamethasone

Human embryonic kidney cells (293) were obtained from the UCSF Cell Culture Facility (Mission Bay) and stable cell-lines were generated as previously described [7]. The purification tags used in this case were streptavidin and calmodulin binding protein. The presence of a hemagluttin (HA) tag was exploited to check for protein expression. Prior to tandem affinity purification, 15 15 cm plates with stable cells at 75–90% confluency were treated for 15 min with 100 nm dexamethasone. For crosslinking, cells were harvested at the appropriate duration and were resuspended in 30 ml of 1.5 mM fresh DSP in PBS (DSP was obtained from Pierce cat. no. 22586). Incubation on ice for 2 hours, with gentle swirling from time to time followed. Tris (pH 7.5 or 8) to a final concentration of 50 mM was added, and cells were incubated on ice for 30 minutes. Cells were pelleted, and washed 2 times with ice-cold PBS to get rid of excess reagent. Lysis and purification of complexes proceeded as described in [7]. To reverse crosslinker, final eluates were reduced in 20 microliters of 50 mM (final) DTT in 50 mM ammonium bicarbonate for 75 minutes at 37°C prior to trypsinization for MS/MS analysis.

### Tandem Mass Spectrometry

TAP-Tagged proteins were eluted and subjected to reduction with DTT and alkylation with iodoacetamide in-gel digestion with trypsin overnight at pH 8.3. Reversed-phase microcapillary liquid chromatography tandem mass spectrometry (LC-MS/MS) was used to identify peptide sequences using a splitless nanoflow EASY-nLC (Proxeon Biosystems, Odense, Denmark) with a self-packed 75 µm-id×15-cm C_18_ column coupled to a hybrid linear ion trap LTQ Orbitrap XL mass spectrometer (Thermo Fisher Scientific, San Jose, CA) in data-dependent acquisition and positive ion mode at 300 nL/min. MS/MS spectra collected via collision-induced dissociation (CID) were searched against the concatenated target and decoy (reversed) Swiss-Prot protein databases using Sequest [Proteomics Browser Software (PBS), Thermo Fisher Scientific] with differential modifications for Met oxidation (+15.99) and fixed modification for Cys alkylation (+57.02). Protein groups were identified if they contained at least two unique peptide sequences from the target database and a ™Sf score >1. False discovery rate (FDR) was calculated to be >1% based on reversed database hits. Single ungrouped sequences were identified if they had Sf scores greater than 0.85 and passed the 1% FDR threshold. Proteins were semi-quantified between experimental conditions using spectral counting and subtractive analyses.

### Cell Based Kinase Assays

HEK cells were transiently transfected with cDNA in the 6-well format at 50% confluency. The next day, the cells were incubated for 1 h in phosphate-free DMEM media (Invitrogen) after which 166 µCi of P^32^ inorganic orthophosphate was added to each well. The cells were lysed 2 h later for immunoprecipitation and Western Blot analysis. 1 µg of pGLUE constructs, 100 ng of GFP and GFP tagged kinases were transfected where stated.

### Reporter Assays

HEK cells in 24-well plates were transfected with a master mix of 10 ng of M34-luc, 5 ng of renilla, 25 ng of GFP. 25 ng of *BMAL* and *CLOCK* and 3.3 pmols of siRNA were also transfected as indicated. Cells were harvested 24 h later and assayed using Promega's Dual Luciferase Assay Kit.

*Stealth human siRNAs* were obtained from Invitrogen (please contact corresponding author for sequence details if desired)

Control Medium GC #2: Forward/Reverse Cat no. 12935112

PHB2: Forward/Reverse Cat no. 10620318/9 PHB2HSS117604 & 5

CK1δ: Forward/Reverse Cat no. 10620318/9 CSNK1DHSS102382

CK1ε: Forward/Reverse Cat no. 10620318/9 CSNK1EHSS102385

PER2: Forward/Reverse Cat no. 10620318/9 PER2HSS113092

STIP1: Forward/Reverse Cat no. 10620318/9 CSNK1EHSS102385

GNBL1L2: Forward/Reverse Cat no. 10620318/9 GNBL1L2HSS115921

SNX24: Forward/Reverse Cat no. 10620318/9 SNX24HSS120748

CRY2: Forward/Reverse Cat no. 10620318/9 CRY2HSS102310

CXORF39: Forward/Reverse Cat no. 10620318/9 CXORF39HSS151899

FAM83H: Forward/Reverse Cat no. 10620318/9 FAM83HHSS138850

IRS4: Forward/Reverse Cat no. 10620318/9 IRS4HSS112348

PHB1: Forward/Reverse Cat no. 1299003 PHB HSS182281 & 2 & 3

### Lumicycle Assays

U2OS cells stably expressing luciferase driven by the *BMAL1* promoter were transfected with siRNA prior to synchronization and bioluminescence was then monitored over several days. Dexamethasone was used to synchronize the cells 24 h after transfection. Period lengths from Day 1 and 2 were determined in plates with good oscillation using LumiCycle Analysis software.

### RT-qPCR

#### Human

We quantified *PER2*, *PHB2* and *GAPDH* (internal control for normalization) transcripts from cells transfected with control, *PHB2*, *CK1δ*, *CK1ε* and *PER2* siRNAs. Total RNA was isolated from HEK cells transfected with siRNA (6.6 pmol in a 24-well plate format) using RNAeasy Mini Qiagen Kit. Reverse CDNA was made using Invitrogen SuperScript III kit. Absolute quantification was determined using PCR standards and carried out with ABI Sybr Green/ROX with gene specific primers. Data was collected and analyzed with ABI 7900HT machine and software, respectively. *GAPDH* was used as an internal control.

Primer sequences:

*PER2* Forward 5′ GGAGAGCCGAAATTTGTAA 3′ Reverse 5′ CCAAGTACCTGATGACGCT 3′
*PHB2* Forward 5′ GGCCCAGGTATCCCTGTT 3′ Reverse 5′ GGCCATCGTCTTGGAGATA 3′
*GAPDH* Forward 5′ CCACCCATGGCAAATTCCAT 3′ Reverse 5′ TCTAGACGGCAGGTCAGGTC 3′

#### *Drosophila*

RNA was isolated by the same method described above from 30–60 fly heads per genotype.

*dphb1* Forward 5′ GGCTGCTCAGTTCTTTAA 3′ Reverse 5′ CATCTCACGCT 3′
*dphb2* Forward 5′ GCACACAGCAAATTGAA 3′ Reverse 5′ GGGATACCTGCTGACGCT 3′
*rpl32* Forward 5′ GCTAAGCTGTCGCACAAAT 3′ Reverse 5′ CGCAGGCCGACCGTTGGGGTT 3′

### PHB2 protein modulation

SiRNA-facilitated KO of *CK1δ* and *CK1*ε mRNA was carried out in HEK cells before PHB2 and ACTIN expression levels were determined by immunoblotting followed by densitometry analysis.

### Fly maintenance, locomotor activity and analysis

Procedures were carried out as described in [3]. Wild-type flies were *w^118^*. RNAi lines were obtained from VDRC. RNAi lines for *dphb2* were line 32362 and 110070. *Dphb1* lines used were 12358 and 12360. For overexpression, hphb2 was cloned into the pUAST construct, injected into larva to make transgenic lines. *Cry*-GAL4 lines were a kind gift from Allada lab.

## Supporting Information

Figure S1 **Gene oscillation and validation of binding/knockdown of PHBs in HEK cells.** (*A*) HEK293 cells can be synchronized with dexamethasone. HEK cells at ∼80% confluency were treated with 100 nM dexamethasone for 2 h and harvested 12, 16, 20, 24, 28, 32, 36, 40 and 44 h later. *Per2* and *Cdk4* transcript levels were quantified using RTPCR. *Per2* transcript levels were normalized to *Cdk4*. *Per2* levels oscillate as previously shown in [6]. (*B*) Western Blot showing that PHB and PHB2 are binding partners. Cells were transfected with HA-tagged PHB and GFP-tagged PHB2 and vice versa. HA tagged proteins were purified using streptavidin beads. (*C*) Graph showing knockdown efficiency relative to control siRNA of *PHB1* and *PHB2* siRNA. Respective siRNAs were cotransfected transiently with HA-PHB or HA-PHB2 and GFP to normalize for transfection and expression into HEK cells. Lysates were analyzed by Western Blot and densitometry was utilized to quantify band intensity.
(TIF)

Figure S2 ***dPHB*** **s are not circadianly-regulated genes and RNAi knockdown efficiency.** (*A*) RTPCR showing *dphb* and *dphb2* transcripts from fly heads in constant darkness. (*B*) RTPCR results showing transcript levels of *dphb* in two phb RNAi lines (phb1rnai1 and phb1rnai2) and two phb2 RNAi lines (phb2rnai1 and phb2rnai2). (*C*) RTPCR results showing transcript levels of *dphb2* in two phb RNAi lines (phb1rnai1 and phb1rnai2) and two phb2 RNAi lines (phb2rnai1 and phb2rnai2).
(TIF)

Figure S3 **Adult locomotor activity actograms and** ***chi-squared*** **periodograms (** ***insets*** **) of control flies (** ***left*** **) and those expressing** ***UAS-dphb2RNAi*** **(** ***middle*** **) and** ***UAS-dbt*** **(** ***right*** **).** To test the role of *PHB2* on circadian regulation *in vivo*, we utilized RNAi expression to knock-down *Drosophila* PHB2 (*dphb2*, (CG15081)). Due to the essential role for dprohibitins in early development, we drove expression only in *cryptochrome*(*cry*)-expressing circadian neurons [16]. (**A**) *UAS-DCRII;Cry16-GAL4* driven expression. When we monitored locomotor activity of adult flies with one copy of *UAS-Dicer II* (*UAS-DCRII*) and *Cry16-GAL4*, their period length was 25.4 h±0.1 h (Fig. S3A, *left* and Table S2). As has previously been shown [3], overexpressing *double-time* (*dbt*), an orthologue of CK1 (NP_733414), results in a longer period length (26.5 h±0.2 h) (Fig. S3A, right). We found that RNAi facilitated knock-down of *dphb2* but not *dphb1* led to period arrhythmicity in a majority of flies tested (Fig. S3A *middle*, Table S2). *Dphb1* (Fig. S2B) and *dphb2* (Fig. S2C) mRNA levels were decreased following the expression of their respective RNAi. (**B**) *Cry39-GAL4* driven expression. Furthermore, knock-down of *dphb2* using a *Cry39-GAL4* driver (Table S2) resulted in flies exhibiting a significantly longer period length (25.2 h±0.2 h) compared to control (24.4 h±0.1 h, p<0.001). (**C**) RTPCR showing the overexpression of human *PHB* and *PHB2* in fly heads using GAL4/UAS system. Since driving expression of a second available *phb2* RNAi line (*UAS-dphb2RNAi2*) did not lead to a decrease in *dphb2* mRNA (Fig. S2C) or show any phenotype (data not shown), we chose to rescue the phenotype elicited in *Cry39-GAL4/UAS-dphb2RNAi* flies by overexpressing human *PHB2*. Overexpressing *hPHB2* partially rescued the period length of *Cry39-GAL4/UAS-dphb2RNAi* flies (Table S2).
(TIF)

Table S1 **CK1δ and CK1ε proteomics Mass Spectrometry results.**
(XLS)

Table S2 **PHB2 function in fly locomotor activity assays.** Adult fly locomotor activity results showing period lengths and rhythmicity. Expression of human transcripts are shown in Fig. S3C.
(XLSX)
