# Supplementary figures and images for: Casein Kinase 1 Proteomics Reveal Prohibitin 2 Function in Molecular Clock
Source: PLoS One. 2012 Feb 27;7(2):e31987. doi: 10.1371/journal.pone.0031987 (PMC3288064; doi:10.1371/journal.pone.0031987)

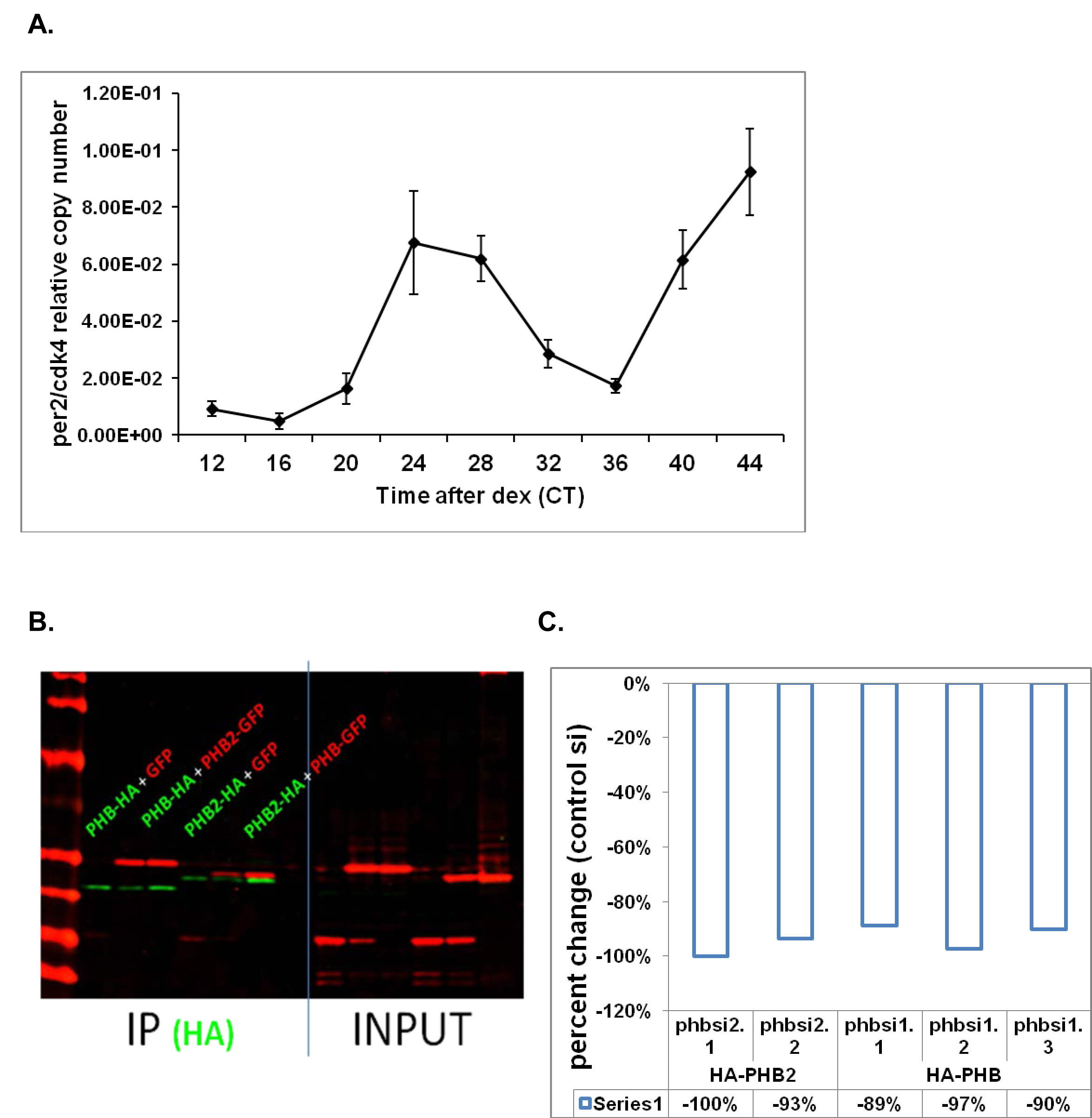

Supplement: Figure S1 — Gene oscillation and validation of binding/knockdown of PHBs in HEK cells. (A) HEK293 cells can be synchronized with dexamethasone. HEK cells at ∼80% confluency were treated with 100 nM dexamethasone for 2 h and harvested 12, 16, 20, 24, 28, 32, 36, 40 and 44 h later. Per2 and Cdk4 transcript levels were quantified using RTPCR. Per2 transcript levels were normalized to Cdk4. Per2 levels oscillate as previously shown in [6]. (B) Western Blot showing that PHB and PHB2 are binding partners. Cells were transfected with HA-tagged PHB and GFP-tagged PHB2 and vice versa. HA tagged proteins were purified using streptavidin beads. (C) Graph showing knockdown efficiency relative to control siRNA of PHB1 and PHB2 siRNA. Respective siRNAs were cotransfected transiently with HA-PHB or HA-PHB2 and GFP to normalize for transfection and expression into HEK cells. Lysates were analyzed by Western Blot and densitometry was utilized to quantify band intensity. (TIF) [file pone.0031987.s001.tif]

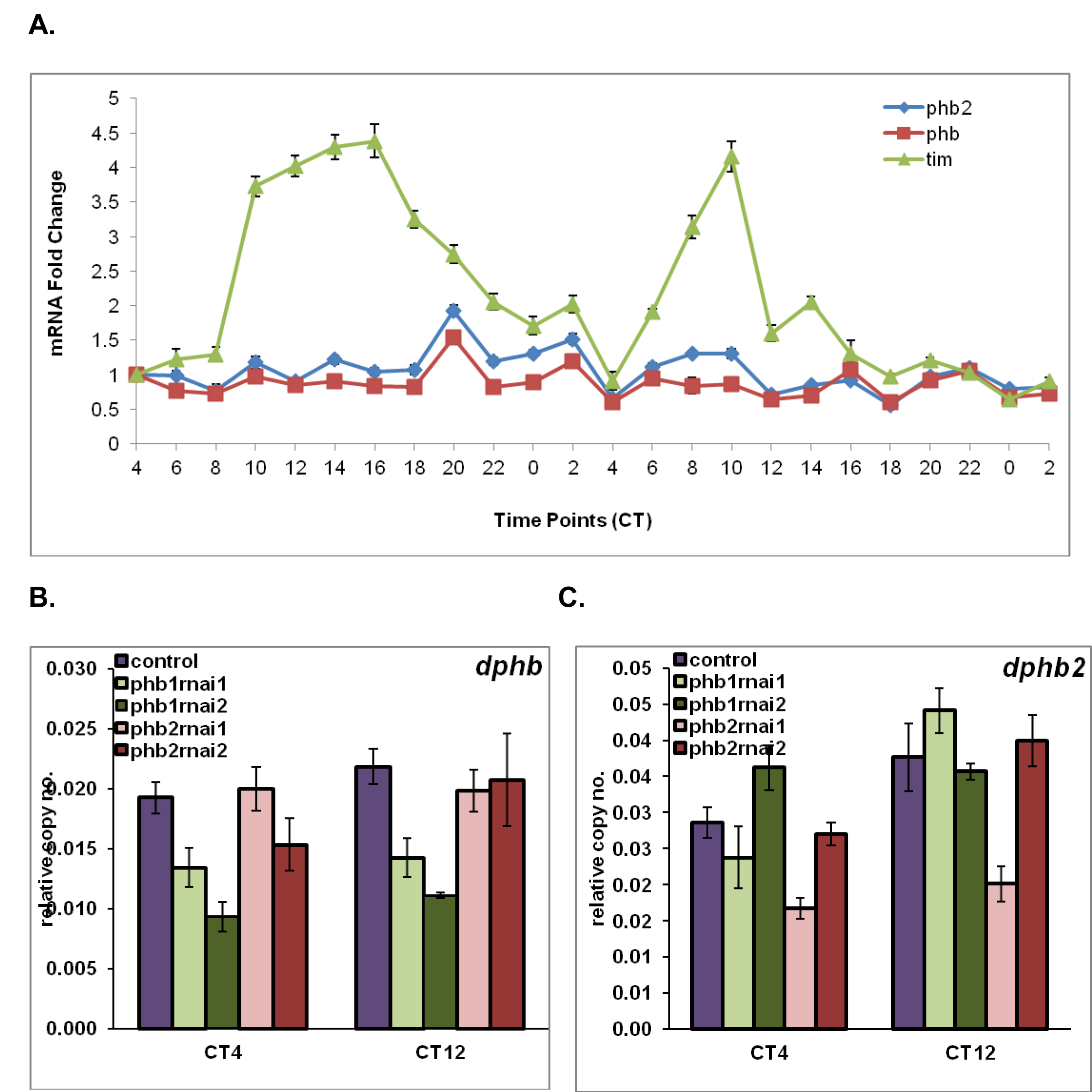

Supplement: Figure S2 — dPHB s are not circadianly-regulated genes and RNAi knockdown efficiency. (A) RTPCR showing dphb and dphb2 transcripts from fly heads in constant darkness. (B) RTPCR results showing transcript levels of dphb in two phb RNAi lines (phb1rnai1 and phb1rnai2) and two phb2 RNAi lines (phb2rnai1 and phb2rnai2). (C) RTPCR results showing transcript levels of dphb2 in two phb RNAi lines (phb1rnai1 and phb1rnai2) and two phb2 RNAi lines (phb2rnai1 and phb2rnai2). (TIF) [file pone.0031987.s002.tif]

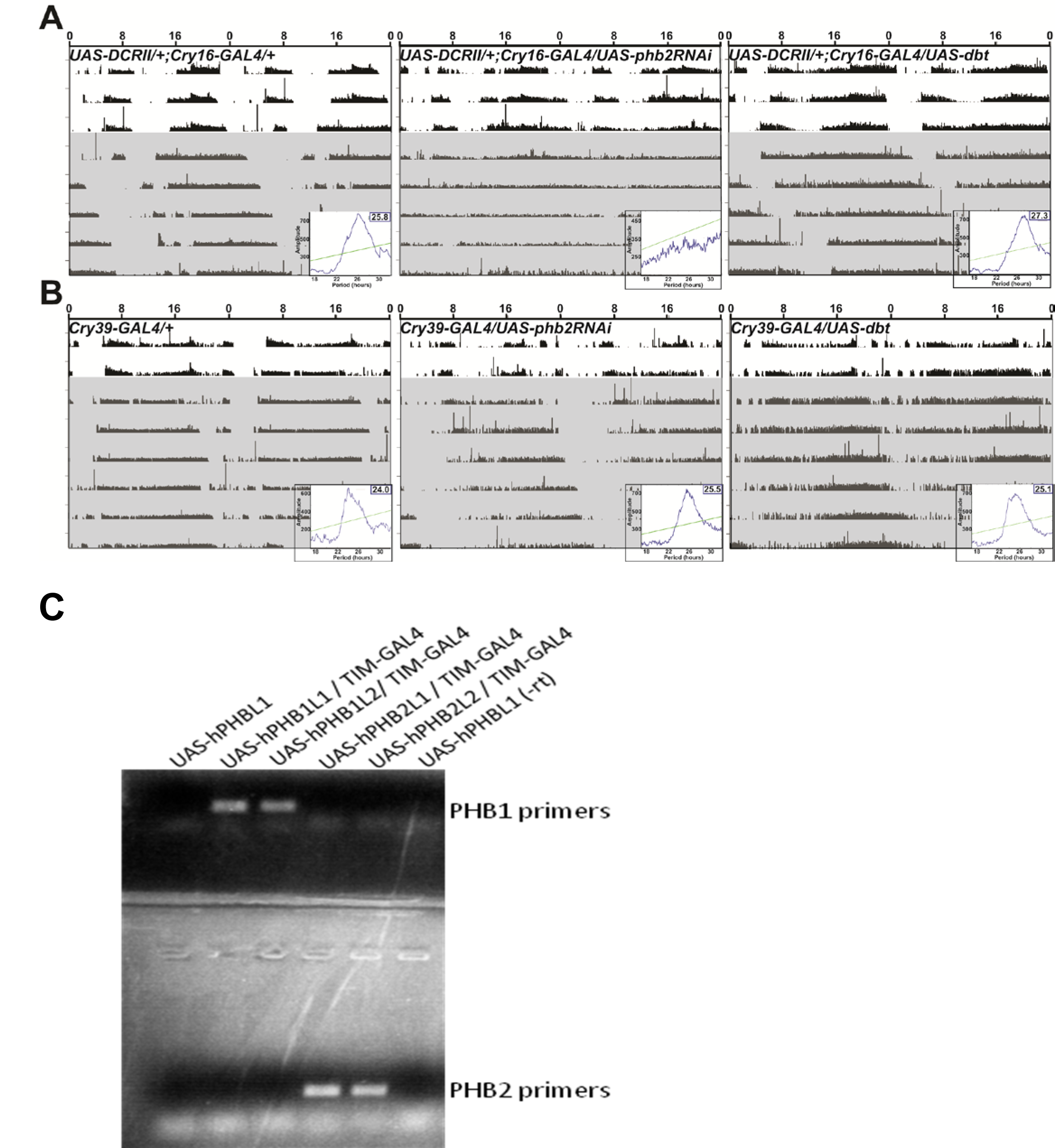

Supplement: Figure S3 — Adult locomotor activity actograms and chi-squared periodograms ( insets ) of control flies ( left ) and those expressing UAS-dphb2RNAi ( middle ) and UAS-dbt ( right ). To test the role of PHB2 on circadian regulation in vivo, we utilized RNAi expression to knock-down Drosophila PHB2 (dphb2, (CG15081)). Due to the essential role for dprohibitins in early development, we drove expression only in cryptochrome(cry)-expressing circadian neurons [16]. (A) UAS-DCRII;Cry16-GAL4 driven expression. When we monitored locomotor activity of adult flies with one copy of UAS-Dicer II (UAS-DCRII) and Cry16-GAL4, their period length was 25.4 h±0.1 h (Fig. S3A, left and Table S2). As has previously been shown [3], overexpressing double-time (dbt), an orthologue of CK1 (NP_733414), results in a longer period length (26.5 h±0.2 h) (Fig. S3A, right). We found that RNAi facilitated knock-down of dphb2 but not dphb1 led to period arrhythmicity in a majority of flies tested (Fig. S3A middle, Table S2). Dphb1 (Fig. S2B) and dphb2 (Fig. S2C) mRNA levels were decreased following the expression of their respective RNAi. (B) Cry39-GAL4 driven expression. Furthermore, knock-down of dphb2 using a Cry39-GAL4 driver (Table S2) resulted in flies exhibiting a significantly longer period length (25.2 h±0.2 h) compared to control (24.4 h±0.1 h, p<0.001). (C) RTPCR showing the overexpression of human PHB and PHB2 in fly heads using GAL4/UAS system. Since driving expression of a second available phb2 RNAi line (UAS-dphb2RNAi2) did not lead to a decrease in dphb2 mRNA (Fig. S2C) or show any phenotype (data not shown), we chose to rescue the phenotype elicited in Cry39-GAL4/UAS-dphb2RNAi flies by overexpressing human PHB2. Overexpressing hPHB2 partially rescued the period length of Cry39-GAL4/UAS-dphb2RNAi flies (Table S2). (TIF) [file pone.0031987.s003.tif]
